# Editorial: The role of GPCRs in obesity

**DOI:** 10.3389/fendo.2024.1404969

**Published:** 2024-04-05

**Authors:** Chunye Zhang, Yi Wang, Takefumi Kimura, Ahmad H. Al-Mrabeh

**Affiliations:** ^1^ Bond Life Sciences Center, University of Missouri, Columbia, MO, United States; ^2^ Molecular Metabolism and Ageing Laboratory, Baker Heart and Diabetes Institute, Melbourne, VIC, Australia; ^3^ Department of Medicine, Division of Gastroenterology and Hepatology, Shinshu University School of Medicine, Matsumoto, Japan; ^4^ Centre for Cardiovascular Science, Queen’s Medical Research Institute, University of Edinburgh, Edinburgh, United Kingdom

**Keywords:** GPCR, obesity, metabolic abnormality, genetic variation, inflammation

G-protein coupled receptors (GPCRs) are the largest superfamily of membrane receptor proteins comprised of four subfamilies, including classes A (rhodopsin), B (secretin and adhesion), C (glutamate), and F (Frizzled) subfamilies (1). The shared common feature of GPCRs is the seven transmembrane segment’s structure, with the amino terminus as the extracellular part and the carboxyl terminus as the intracellular part. GPCRs are responsible for recognizing and interacting with a variety of extracellular signaling mediators such as metabolites, cytokines, ions, neurotransmitters, as well as hormones (2). The extracellular terminus of GPCRs recognizes and interacts with small molecules, ligands, and proteins, resulting in the conformational change of GPCR, which leads to the activation of the intracellular signaling pathway. Therefore, they play a pivotal role in the regulation and mediation of a variety of cellular and molecular mechanisms and signaling pathways that are closely associated with human health and diseases such as cancer, autoimmune and neurogenerative diseases, diabetes, and other metabolic diseases (3–7). GPCRs serve as ideal therapeutic targets for many diseases. To date, the GPCRs-targeted therapeutic medicine accounts for about 35% of the approved drugs by the US Food and Drug Administration (FDA) (8).

Obesity is a complicated chronic health condition defined as BMI ≥ 30 Kg/m^2^. It develops after long-term positive calorie balance causing excessive expansion of adipose tissues and ectopic fat accumulation. Obesity impacts the health of both adults and children and increases the risk of other diseases such as cardiovascular disease, type 2 diabetes, stroke, and cancer (9). GPCRs, directly or indirectly, regulate most metabolic processes including glucose and energy homeostasis, both of which are critical in obesity development and progression. Therefore, investigation of the significant role of GPCRs in obesity attracts extensive attention for dissecting disease pathogenesis, identifying therapeutic targets, and improving the treatment efficacy (1, 10).

This Research Topic focuses on the most recent discovery made in the field of GPCRs and obesity, such as advances in genetic variation findings, genetic variant association, functional studies of GPCR variants involved in obesity development and progression, signaling pathways and mechanisms of GPCRs involved in the development of obesity, innovative methods, model systems for investigating GPCRs in clinical and preclinical studies, and GPCRs as therapeutic targets for obesity. Four articles have been published on this topic, including three original research articles and one insightful review.

The original research conducted by Michałowska et al. aimed to reveal the association between genetic variation of the glucagon-like peptide 1 receptor (GLP1R) gene and obesity, as well as metabolic health. In this study, researchers characterized the body mass, metabolic syndrome, anthropometric factors, and other metabolic parameters and their association with single nucleotide variants of the GLP1R gene (rs2268641 and rs6923761). They found that AA carriers of rs6923761 were associated with excessive weight and higher glucose concentration compared to the GC variant carriers. Meanwhile, for rs2268641, TT carriers showed lower frequency in the group with excessive body weight. Overall, this study not only reveals GLP1R genetic association with the susceptibility of the body mass but also inspires other researchers in the field to further the investigation on the underlying mechanism of how the discovered variants related to the increase of body mass. Furthermore, since this study was conducted in a Polish cohort, the larger-scaled data from the general population are desired to get a full understanding of this association. Most importantly, as the authors and other researchers pointed out, the variants of GLP1R may contribute to the disease risk, as well as the treatment efficiency when using GLP1R agonist therapy in obese patients. This has driven the perspective research direction.

The original research performed by Bloyd et al. focused on the investigation of genetic variants in cAMP-dependent protein kinase (PKA) signaling pathway-linked genes among youth cohorts of obese or abnormal metabolic health conditions. The PKA pathway plays an essential role in regulating metabolism and energy balance. This research aimed to examine and reveal the association between genetic loci among pediatric patient cohorts classified as different ethnic groups. The study showed that 49 confirmed variants associated with the PKA signaling pathway have been identified. Among them, 29 variants meet the criteria as high-frequency variants. This study sheds light on the importance of the early detection of genetic contribution and obesity susceptibility, which is important for early intervention and prevention of high-risk individuals or groups.

GPCR, the purinergic P2Y_2_ receptor (P2Y_2_R), plays a critical role in glucose homeostasis and inflammation. Meanwhile, accumulating studies have demonstrated the sex-associated difference between males and females regarding insulin tolerance, glucose metabolism, and immune response. However, less data is available related to the sex difference in the influence of P2Y_2_R in glucose regulation and inflammation. To fill this gap, Ulbricht et al. conducted original research to address the sex variation in the effect that P2Y_2_R played in the regulation of glucose homeostasis, as well as inflammation. The results of this research demonstrated that the P2Y_2_R plays a significant impact on glucose homeostasis (both at the fasting level and LPS-induced inflammation) in males compared to females. This study not only addresses the sex-dependent effect of P2Y_2_R in glucose regulation but also provokes curiosity regarding the underlying mechanism from the perspective of systematical evaluation. Moreover, the result raises the awareness of considering sex-specificity when using a pharmaceutical treatment that is associated with purinergic signaling, such as P2Y_2_R agonists.

A high-quality review provided by Pocai focused on the discussion of the hot topic related to the treatment and weight management of obesity. The author centered the discussion on the breakthrough of GPCR-targeted therapies, such as FDA-approved GLP1R agonist as an anti-obesity medication. This event accelerated a surge of a great number of new players participating in the exploration of anti-obesity medications and therapeutic strategies. The most significant progress has been discussed by the author, such as glucagon receptor (GCGR), glucose-dependent insulinotropic polypeptide receptor (GIPR), dual agonists for GLP-1/GIP and GLP-1/GCGR, triagonist peptides of the GLP-1R, GIPR and GCGR. Furthermore, the review also discussed the GPCR-targeted treatment related to energy homeostasis regulation such as 5-hydroxytryptamine receptor 2C (5-HT2CR), Ghrelin receptor (GHSR), G protein-coupled receptor 40 (GPR40). The author concluded with up-to-date insights into the anti-obesity therapeutic strategy; meanwhile, the author also emphasized the importance of some aspects that need to be considered, such as monitoring the cardiovascular system related to dual agonists or triagonist peptides treatment, evaluation of off-target toxicities related to oral small molecules.

In summary, the three original research and one review articles on this Research Topic shed some light on the importance of GPCRs-related signaling pathways, underlying mechanisms, and therapeutic potential in the treatment of obesity. In general, the research field related to the GPCR in obesity attracts researchers from both pre-clinical and clinical practice. Further basic science and clinical studies are desired to fully understand the underlying mechanism of obesity and associated cardiometabolic diseases to develop novel therapies. Meanwhile, studies that aim to explore new strategies to enhance the efficacy and safety of the developed targets are needed to facilitate the translation of findings from pre-clinical models to clinical trials.

## Author contributions

CZ: Conceptualization, Data curation, Writing – original draft, Writing – review & editing. YW: Writing – review & editing. TK: Writing – review & editing. AHM: Writing – review & editing.
